# Fire Resistance Evaluation of New Wooden Composites Containing Waste Rubber from Automobiles

**DOI:** 10.3390/polym14204465

**Published:** 2022-10-21

**Authors:** Vladimír Mancel, Iveta Čabalová, Jozef Krilek, Roman Réh, Martin Zachar, Tereza Jurczyková

**Affiliations:** 1 Department of Environmental and Forestry Machinery, Faculty of Technology, Technical University in Zvolen, Študentská 26, 960 01 Zvolen, Slovakia; 2 Department of Chemistry and Chemical Technologies, Faculty of Wood Sciences and Technology, Technical University in Zvolen, T. G. Masaryka 24, 960 53 Zvolen, Slovakia; 3 Department of Mechanical Wood Technology, Faculty of Wood Sciences and Technology, Technical University in Zvolen, T. G. Masaryka 24, 960 53 Zvolen, Slovakia; 4 Department of Fire Protection, Faculty of Wood Sciences and Technology, Technical University in Zvolen, T. G. Masaryka 24, 960 53 Zvolen, Slovakia; 5 Department of Wood Processing, Czech University of Life Sciences in Prague, Kamýcka 1176, Suchdol, Prague 6, 16521 Prague, Czech Republic

**Keywords:** waste rubber recycling, wooden composites, flammability, spontaneous ignition temperature, mass burning rate, calorific value

## Abstract

Particleboards containing waste rubber (tires and mixtures of isolators and carpets) filler were evaluated from the point of view of its flammability. The assessment of the utilization of these composites in the construction industry was analyzed through the determination of their spontaneous ignition temperatures, mass burning rate and calorific value. Based on the results of spontaneous ignition temperatures, similar values between particleboards and particleboards containing 10%, 15% and 20% of waste tires were obtained. The average time was from 298 s to 309 s and the average temperature was from 428.1 °C to 431.7 °C. For the mass burning rate, there were similar results between particleboards and particleboards containing 10% of waste tires and waste rubber. The time to initiation was 34 s and the time to reaching a maximal burning rate was from 66 s to 68 s. The calorimetry results showed similar properties for the calorimetric value and ash content in particleboards and particleboards containing 10% of waste tires and waste rubber. The calorific value was from 18.4 MJ·kg^−1^ to 19.7 MJ·kg^−1^ and the ash content from 0.5% to 2.9%.

## 1. Introduction

The continuous development of the industry, as well as consumer lifestyles, has led to an increase in the amount of produced rubber waste. This waste degrades very slowly and, therefore, remains part of the environment for a long time. The amount of rubber-based waste is constantly increasing, related to the increase in the amount of tires and the number of used cars. In a paper by Dwivedi et al. [1], they state that, globally, the annual production of tires is at 1400 million units when combining all types, with an estimated 17 million tons of used tires generated every year. Worn tires and other types of waste rubber (isolations, carpets, etc.) from automobiles are a big global problem. The content of this kind of waste is still much higher than the amount of waste that can be rationally assessed. According to Formela et al. [2], approximately 1200 million tons of tires are predicted to be discarded every year by 2030. Tires are composed of synthetic polymers (46–48%), such as polyamide, butyl rubber, butadiene rubber and styrene–butadiene rubber [3,4]. Carbon black is added to the rubber to improve abrasion resistance [5]. Silica, together with carbon black, is the second major component of tires [6]. Based on the paper by Danon and Gorgens [7], the most common constituents of tires are natural rubber (14–27%); synthetic rubber (14–27%); fillers such as silica (26–28%); sulfur (5–6%); plasticizers based on oils and resins (5–6%); steel and textile cords, etc.

There are only a few options for obtaining original-quality rubber or other similar rubber-based products. The secondary use of tires is, for example, as a part of flooring, for noise barriers [4], energy recovery [4,8], etc. One of the options for recycling rubber is crushing it into a powder, which is then, subsequently, mixed with thermoplastic resins. This process produces thermoplastic elastomer (TPE) compounds, which are multifunctional polymer materials. Their advantageous properties are their elasticity and good processing [9]. Another way to valorize rubber waste is to use it as part of composites. Wood–plastic composites are materials with many advantageous properties that are composed of natural (wood) and synthetic polymers. Such composites can be used in the construction as well as infrastructure and transportation industries [10]. The preparation of composite materials is accomplished by the mixing of wood particles with a synthetic polymer at a high temperature, ranging from 170 °C to 200 °C. At such high temperatures, some problems can occur, e.g., the extractive compounds from wood can migrate to its surface. This can manifest through interaction reductions between the synthetic polymer, the wood and the used binder, and can be the consequence of a decrease in the composites’ mechanical properties [11]. Mechanical properties of composites also depend on the weight fraction of components, the type of additives, used pressing temperature, etc. [12,13,14,15,16,17]. By adding a synthetic polymer to the wooden matrix, some physical properties of particleboards can be improved. According to Ayrilmis et al. [18], the water resistance of the boards is improved with the increase in the rubber crumb/wood particle ratio. In the manufacturing of wood-based composites, it is better to find the most optimal conditions for the preparation of composites, such as pressing time and temperature, amount of resin, board density, etc. [19]. The flammability of materials containing wood is their big disadvantage. Wood, as a natural material, is composed primarily of organic substances containing carbon, and these compounds are flammable [20]. The flammability of polymers depends on the content of carbon and hydrogen in their macromolecules, which ranges from over 85% of C and 14% of H to 0% of hydrogen, e.g., in inflammable nitrozofluoric polymers [21]. The most important properties of flammable materials are their heat release rate, time to ignition, extinction flammability index and thermal stability index, surface spread of flame and fire resistance, mass loss, smoke toxicity and limiting oxygen index [22,23]. Analytical and finite element-based models have been developed to predict the response of polymer composites to high temperatures and fire [23]. According to Babrauskas [24], ignition depends on various interrelated factors. As the surface is exposed to a heat flux, initially, most of the heat is transferred into the inside of the specimen. The rate of this heat transfer is dependent on the material’s thermal properties encompassing the ignition temperature, material thermal conductivity, material specific heat and the density of the material.

Several researchers have evaluated the flammability of wood-based composite materials [20,22,23,24,25]. According to Harada et al. [26], for the utilization of wood-based boards as a structural material, the following qualities are desired for fire safety:

- The structure does not deform, melt or break down.
- The unexposed side’s temperature does not exceed the burning temperature of the combustible material.
- The structure does not crack or otherwise become damaged by fire outside the building.

This paper follows up on our previous research in the field on the use of plastic and rubber waste from automobiles, and the possibilities of its recycling. The aim of this study is to use these wastes (in proportions of 10%, 15% and 20%) as a part of particleboards. Wood-based material burning in buildings poses a risk. When using these materials as a structural element in buildings or as part of furniture, it is necessary to assess them from the perspective of fire safety. For this reason, the flammability of wood–rubber composites was analyzed through the determination of their spontaneous ignition temperature, mass burning rate and calorific value. The results could be useful in evaluating the use of these composites, from the perspective of fire protection properties in the construction industry, predominantly in the interior.

## 2. Materials and Methods

### 2.1. Material

#### 2.1.1. Rubber Waste

**Figure 1:**
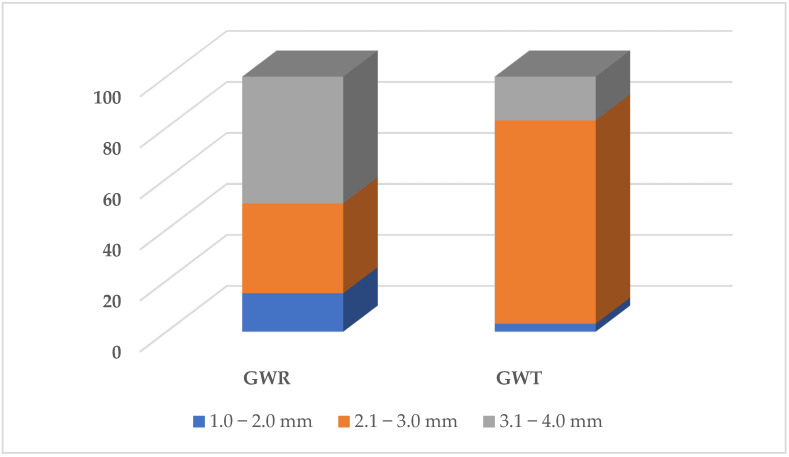
Size distribution of used rubber waste (GWR, GWT).

The size of the granulate from waste rubber—“GWR” (carpets and isolators)—was from 1.0 to 4.0 mm (size distribution in Figure 1). The granulate consisted of a mixture of processed materials, including polyester, glass fibers, polyurethane, paper and EPDM (ethylene propylene diene monomer).

The size of the granulate from waste tire—“GWT”—was from 1.0 to 4.0 mm (size distribution in Figure 1). The granulate consisted of the processed material SBR (styrene–butadiene rubber).

The granulate from waste rubber and tires from discarded automobiles was produced by AVE SK-Kechnec plant Slovakia [27].

#### 2.1.2. Composite Processing

In the experiment, wood particles prepared from fresh spruce logs were processed by the company Kronospan s.r.o., Zvolen, Slovakia, and obtained from them. The dimensions of particles commonly used for the core layer and selected for the single-layer particleboard production were from 0.25 to 4.0 mm. Particles were dried to a moisture content of 4%.

A commercially available UF resin Kronores CB 1100 F (Diakol Strážske s.r.o., Strážske, Slovakia) was used for pressing single-layer particleboards with the addition of crushed rubber. The European standard EN 309 [28] defines particleboards as a “panel material manufactured under pressure and heat from particles of wood (wood flakes, chips, shavings, saw-dust and similar) and/or other lignocellulosic material in particle form (flax shives, hemp shives, bagasse fragments and similar), with the addition of an adhesive”. The adhesive that was used to bond the wood particles and crushed rubber had a solid content of 67.1%, viscosity of 460 mPa·s, condensation time of 55 s and a pH value of 8.6. Ammonium nitrate NH_4_NO_3_ (47%) was added to the adhesive mixture as a hardener. Paraffin, used as a 35 wt% water emulsion, was applied to the particles in amounts of 0.6%. Such a composition of the adhesive mixture was added to particles in an amount of 11 wt%. The used material was crushed rubber, which was mentioned in the previous chapter.

**Figure 2:**
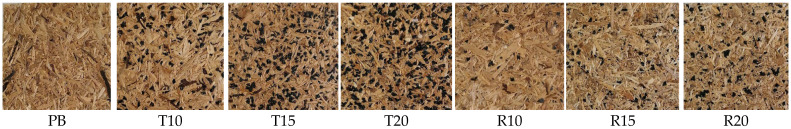
Samples of composites used for the determination of spontaneous ignition temperatures (20 × 20 × 15 mm, length × width × thickness).

The single-layer particleboard with the addition of crushed waste rubber had the dimensions of 360 × 280 × 15 mm, and was prepared in laboratories of the Technical University in Zvolen, Slovakia. The moisture content of the particles mixed with UF resins was 9.5%. A particleboard was prepared using common technology, i.e., firstly, particle mats were cold-prepressed at 1 MPa, followed by hot pressing in pressure (CBJ 100–11 laboratory press, TOS, Rakovník, former Czechoslovakia) with a maximum temperature of the pressing plates in the press of 230 °C, a maximum pressing pressure of 6.50 MPa and a total pressing time of 356 s, which had to be longer than in the conventional production of all-wood particleboards due to the presence of crushed waste rubber [29]. Six boards of each condition were produced with the average density ranging from 554 to 615 kg·m^3^ (Figure 2, Table 1). For the composite characterization, the bending strength (MOR) test was performed on ten replicates per sample according to EN 310 [30], as well as the water absorption and thickness swelling tests after 2 and 24 h according to the STN EN 317 standard [31]. Measurements of physical properties were carried out on six samples per composite. The principle was to place samples into water and to record the thickness and weight after 2 and 24 h (Table 2).

### 2.2. Methods

#### 2.2.1. Spontaneous Ignition Temperatures

One of the fire-technical properties expressing the material’s resistance to burning is the spontaneous ignition temperature (SIT). The SIT was determined according to the STN ISO 871 standard [32]. The tested samples were heated in a heating chamber. They were subjected to different temperatures without igniting a flame. Using thermocouples (type K) with a diameter of 0.5 mm, the temperature profile in the furnace was recorded. The temperature was recorded using the data logger ALMEMO^®^ 710 (Ahlborn Mess- und Regelungstechnik GmbH, Holzkirchen, Germany). The spontaneous ignition temperature was the lowest air temperature at which the sample was ignited within 10 min. Subsequently, the induction time was determined. Measurements were performed in twenty replicates per sample.

#### 2.2.2. The Mass Burning Rate

Another of the fire-technical properties is the reaction to a fire test, which was determined according to the ISO 11925-2 standard [33]. The mass burning rate was measured using a device consisting of an electronic balance (accuracy of two decimal places), a metal sample holder, a weight, a metal loading frame for placing a radiant heat source and an infrared thermal heater with an input of 1000 W. The principle of the determination was that the sample was placed in the holder at 30 mm from the heat source for a certain time of 600 s; then, the weight change was recorded every 10 s. The heat flux of the infrared heat heater was 30 kW∙m^−2^. Measurements were performed in twenty replicates per sample.

To determine the burning rate in the specified time interval, the absolute burning rate *ʋ* was calculated according to the relational Equation (1):

(1) $$\vartheta =\frac{\delta \left(\tau \right)-\delta \left(\tau +\Delta \tau \right)}{\Delta \tau }$$

where:

ϑ—absolute burning rate (%·s^−1^);

*δ* (*τ*)—specimen mass in the time (*τ*) (%);

*δ* (*τ* + Δ*τ*)—specimen mass in the time (*τ* + ∆*τ*) (%);

Δ*τ*—time interval in which the mass values were recorded (s).

The mass burning rate was calculated according to the pattern XY using values of mass losses, which was measured using the laboratory scales Radwag PS 3500.R2 and software Radwag communication.

#### 2.2.3. Calorimetry

The calorific values of the composite samples were determined using a C 200 calorimeter (IKA^®^-Werke GmbH & Co. KGIKA, Staufen, Germany). The results were evaluated using Cal Win software (IKA^®^-Werke GmbH & Co. KG, Staufen, Germany). The measurements were carried out in accordance with the standard STN ISO 1928 (44 1352) [34]. The amount of ash was calculated from the difference in weight between the original sample before combustion and the residue after combustion in the calorimeter. Measurements were performed in four replicates per sample.

## 3. Results and Discussion

### 3.1. Spontaneous Ignition Temperature

Obtained results of the spontaneous ignition temperature, as well as the spontaneous ignition times of the tested composites, are presented in Table 3.

For the highest fire resistance, it was important to observe the longest ignition time and the highest temperature. Very similar results for both the average ignition temperature and time were obtained in composite samples containing waste tire filler (T10, T15 and T20) compared to the particleboards. From the perspective of composite samples containing waste rubber filler (R10, R15 and R20), it was obvious that the time to ignition was higher than both for the PBs and samples containing tire filler, but the average temperature was lower depending on the content of rubber filler. The lower temperature of thermal degradation may have been due to the presence of volatile substances in materials composed of recycled rubber [35]. We could state that the fire resistance (from the point of view of the SIT) of composites containing 10–20% rubber filler was comparable to the properties of conventional particleboards. The wood ignition temperature was between 300 °C and 350 °C under constant irradiation. According to Babrauskas [36], panel products, such as plywood or particleboards, have fire properties very similar to solid wood. Therefore, solid wood results would be applicable to them. Vermesi et al. [37] studied the ignition of fiberboards under transient irradiation. Surface temperatures at ignition were close to 300 °C for the majority of cases, with the exception being the parabolic flux, with a time to peak of 260 s and a peak irradiation of 30 kW∙m^−2^. Tureková et al. [38] also determined the ignition temperature of a sample of OSB (oriented strand board). The samples were placed horizontally and exposed to a heat flux of 43 to 50 kW·m^−2^ using an electrically heated conical radiator. The time to ignition and ignition temperature at the upper and lower surfaces of a 15 mm thick OSB sample corresponded to individual radiant heat flux densities. The determined values ranged from a time to ignition of 142 (s) and temperature of 287 (°C) at a heat flux of 44 (kW∙m^−2^), and to a time to ignition of 58 (s) and temperature of 319 (°C) at a heat flow of 50 (kW∙m^−2^).

### 3.2. The Mass Burning Rate

**Figure 3:**
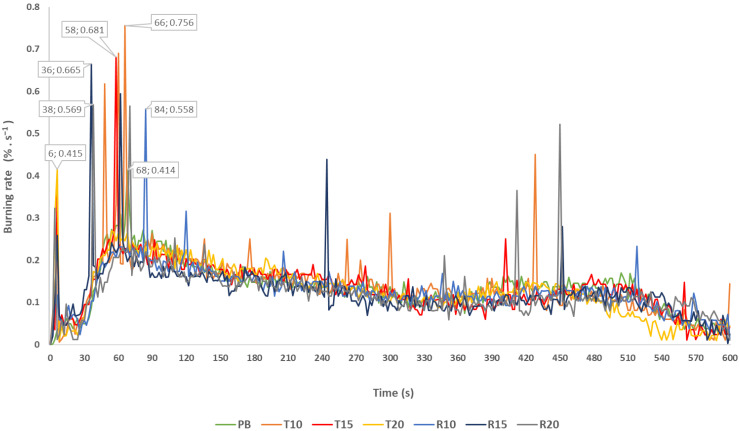
Absolute burning rate of the composite samples.

In Figure 3, there is a graph depicting the results of mass burning, where the values of the maximum burning rate, as well as the time to reach these values of the maximum burning rate, were given depending on a heat flux of 30 kW∙m^−^^2^. The burning rate of the samples was calculated based on weight losses in time intervals of up to 600 s.

Based on Figure 3, showing curved variations of the mass burning rate, it could be stated that the maximum burning rates could be observed within time intervals of 6 s to 84 s, during which the specimens were exposed to thermal loading. From the perspective of the fire protection properties, an important role was played by the time it took for the tested material to reach the given value of flash point. The results of our measurements showed that by changing the content of impurities (tires and rubber), the time to the initiation of composite samples was decreasing.

At the thermic load with a heat flux of 30 kW·m^−^^2^ of PB samples, the time to initiation was 34 ± 0.05 s, the time to reaching a maximal burning rate was 68 s and the maximal burning rate was 0.414%·s^−^^1^. Similar values were recorded for the T10 samples, where the time to initiation was 34 ± 0.12 s, the time to reaching a maximal burning rate was 66 s and the maximal burning rate was 0.756%·s^−^^1^. Based on the determination of the time to initiation 32 ± 0.09 s and the maximal burning rate, the R10 samples reached the highest time for reaching the maximal burning rate, which was 84 s, and the maximal burning rate, which was 0.558%·s^−^^1^. Based on the results in Table 4, the time to initiation and the maximal burning rate were comparable for all samples tested. However, with the increasing percentage of waste tires and waste rubber, there was a shorter time to initiation recorded. This indicated a lower thermal stability of the samples (R15, R20, T15 and T20). Based on the monitored fire-technical parameters, the time to initiation, the maximal burning rate and the time to reaching the maximal burning rate, there was the assumption that the R10 and T10 samples reported almost identical values as the PB samples (samples without an admixture), which indicated their comparable thermal stability.

Spruce, beech and pine wood are among the main materials used for the production of wood composite materials. Several authors evaluated the fire-technical properties of these types of wood. Zachar et al. [39] evaluated the fire-technical properties of spruce wood samples. They reached a temperature of 400 °C for the flammability point in the 550th second, and a flash point of 360 °C in 560 **s**. Zigo et al. [40], in their article, evaluated the minimum temperature of the flammability point of spruce wood samples using different pressures. The values of the monitored property ranged from 470 °C to 520 °C. Similar flash point temperatures (of approximately 487.9 °C) of spruce wood were also reached in the work by Hagen et al. [41]. The inductive period, an interval of 460–560 °C, of spruce pellets was described by Martinka et al. [42]. The flammability point temperature (478 °C) of pine wood was investigated by Delichatsios et al. [43]. Osvaldová et al. [44], by using a conical calorimeter, evaluated the initiation time of different wood samples loaded with a heat flux of 35 kW∙m^−^^2^. They reached comparable results to ours in the initiation time (30 to 54 s) for spruce wood. In the case of spruce wood, they stated that which was comparable to our results. Sultan [45] evaluated the influence of insulation types on the fire resistance of external wall assemblies with OSB cladding. OSB boards are often parts of structural elements in the exterior, and, therefore, it is crucial to carry out extensive fire tests on them.

On the other hand, according to Dan et al. [46], the ignition temperature of tire powders was 349.71 °C. Janowska et al. [21] stated the ignition temperatures of butadiene (BR), butadiene–acrylonitrile (NBR) and butadiene–styrene (SBR) rubbers to range from 335 °C up to 348 °C. In their paper, they described that the significant influence on polymer flammability is exerted by both the rate of mass loss during combustion and the type of products being formed.

A review of the literature showed that the flammability of rubber-based materials was higher compared to wood, which we also confirmed in the presented article.

### 3.3. Calorimetry

Calorimetry and the evaluation of the calorific value are necessary for defining the energetic content of materials or the amount of heat that materials generate on their complete combustion. Based on the results reported in Table 5, it could be stated that the determined calorific values of composites ranged from 18.4 MJ·kg^−^^1^ (PB) to 21.5 MJ·kg^−^^1^ (R20) depending on the content of rubber fillers.

A higher filler content of rubber/recycled tires in particleboards means an increase in the calorific values. The highest values were recorded for the R20 sample. The higher content of ash was recorded in composites containing recycled tire fillers compared to both composites with rubber fillers and particleboards. An increase in the ash content means a reduction in the calorific value of materials [47], which was also confirmed in this work. Comparing the calorific value of particleboards and composites containing rubber fillers, it could be stated that composites with fillers generated more heat. Separated samples of both granulates, rubber (insulation and carpet) and tires, had higher calorific values of 29.9 MJ·kg^−1^ (GWR) and 36.4 MJ·kg^−1^ (GWT). Based on the results of Kunioka et al. [48], the combustion energy of rubbers related linearly to the carbon content of these samples. The calorific value of rubber materials based on the findings of several authors ranged from 22.2 to 31 MJ·kg^−1^ [48,49,50,51]. According to Danon and Gorgens [7], tires were composed of approximately 90% of organic materials and, compared to other rubber materials, contained higher calorific values ranging from 29 to 39 MJ·kg^−1^.

## 4. Conclusions

Particleboards (PBs) are commonly used as the interior lining material in commercial or residential buildings. Due to the flammability of these materials, it is important and necessary to carry out the standard test methods of evaluating the fire resistance of these building materials.

Based on the results of the spontaneous ignition temperatures, the average time of prepared composites ranged from 298 s to 351 s, and average temperature ranged from 414.1 °C to 431.7 °C. For mass burning rate, the time to the initiation of prepared composites ranged from 28 s to 34 s, the maximal burning ranged from 0.414%·s^−^^1^ to 0.756%·s^−^^1^, and the time for reaching the maximal burning rate ranged from 6 s to 84 s. Based on the results of the calorimetry analysis, the calorific values of prepared composites ranged from 18.358 MJ·kg^−^^1^ to 21.5 MJ·kg^−^^1^ depending on the content and type of rubber filler. The calorific value was 29.9 MJ·kg^−^^1^ for rubber (isolators, carpets) and 36.4 MJ·kg^−^^1^ for tires. These granulates also had the highest ash content of 18.2% for rubber and 7.4% for tires. The lowest ash content of 0.6% was found in a particleboard sample without any rubber fillers.

By evaluating the fire-technical properties of particleboards containing rubber waste filler, it could be stated that the fire resistance of these materials was comparable to conventional particleboards. From the point of view of fire-technical properties, particleboards containing rubber waste fillers can be used as a common building material (in wall partitions, as part of furniture, etc.). The burning characteristics data measured in this research could be useful as inputs for fire growth models for predicting fire behavior of special composite materials.

## Figures and Tables

**Table 1:** Composite characterization.

Signification	Composite Characterization
PB	Particleboard
T10	Particleboard—containing 10% of GWT
T15	Particleboard—containing 15% of GWT
T20	Particleboard—containing 20% of GWT
R10	Particleboard—containing 10% of GWR
R15	Particleboard—containing 15% of GWR
R20	Particleboard—containing 20% of GWR

**Table 2:** Composite mechanical and physical properties.

Sample	Bending Strength (MPa)	Water Absorption (%)		Thickness Swelling (%)	
		2 h	24 h	2 h	24 h
PB	0.294 ± 0.052	141.41 ± 8.61	166.16 ± 5.29	59.87 ± 5.50	70.28 ± 6.62
T10	0.211 ± 0.054	73.08 ± 7.05	97.94 ± 4.65	23.35 ± 3.20	30.74 ± 2.40
T15	0.223 ± 0.043	53.36 ± 9.85	88.60 ± 7.08	17.82 ± 2.90	26.57 ± 2.63
T20	0.215 ± 0.049	35.18 ± 3.72	99.58 ± 7.90	21.73 ± 3.09	27.63 ± 2.28
R10	0.284 ± 0.017	89.01 ± 4.12	117.40 ± 3.35	27.82 ± 2.50	32.98 ± 4.63
R15	0.263 ± 0.034	83.98 ± 2.67	113.63 ± 5.64	24.90 ± 1.64	31.47 ± 2.74
R20	0.176 ± 0.040	80.77 ± 7.93	107.33 ± 8.23	25.35 ± 1.55	31.50 ± 2.96

**Table 3:** Spontaneous ignition temperature of composite samples.

Sample	Average Time τ (s)	Average Temperature t (°C)
PB	309 ± 1.2	430.0 ± 1.2
T10	298 ± 1.1	431.7 ± 1.1
T15	299 ± 2.1	428.8 ± 1.3
T20	302 ± 1.7	428.8 ± 2.1
R10	318 ± 1.4	424.8 ± 1.5
R15	342 ± 1.9	415.4 ± 1.8
R20	351 ± 2.4	414.1 ± 1.5

**Table 4:** Values representing burning rate and burning time of the composite samples.

Composite Sample	Value		
	Time to Initiation (s)	Maximal Burning Rate (%·s^−1^)	Time of Reaching Maximal Burning Rate (s)
PB	34 ± 0.05	0.414	68
T10	34 ± 0.12	0.756	66
T15	32 ± 0.08	0.681	58
T20	30 ± 0.03	0.415	6
R10	32 ± 0.09	0.558	84
R15	30 ± 0.15	0.665	36
R20	28 ± 0.07	0.569	38

**Table 5:** Calorific value and ash content of rubber materials and composites.

Sample/Property	Calorific Value (MJ·kg^−1^)	Ash Content (%)
**GWT**	36.4 ± 0.8	7.4 ± 2.4
**GWR**	29.9 ± 0.3	18.2 ± 0.5
**PB**	18.4 ± 0.2	0.6 ± 0.2
**T10**	19.7 ± 0.2	2.9 ± 1.2
**T15**	19.8 ± 0.2	2.4 ± 0.1
**T20**	20.5 ± 0.3	2.9 ± 0.3
**R10**	19.4 ± 0.1	0.5 ± 0.2
**R15**	20.2 ± 0.3	1.2 ± 0.4
**R20**	21.5 ± 0.6	1.4 ± 0.5

## Data Availability

The data presented in this study are available on request from the corresponding author.
